# Care cascade of tuberculosis infection treatment for people living with HIV in the era of antiretroviral therapy scale-up

**DOI:** 10.1038/s41598-022-20394-2

**Published:** 2022-09-27

**Authors:** Kuan-Yin Lin, Chia-Jui Yang, Hsin-Yun Sun, Yuan-Ti Lee, Bo-Huang Liou, Ing-Moi Hii, Tun-Chieh Chen, Sung-Hsi Huang, Chun-Yuan Lee, Chin-Shiang Tsai, Chi-Ying Lin, Chun-Eng Liu, Hsi-Yen Chang, Chien-Yu Cheng, Po-Liang Lu, Chien-Ching Hung, Taiwan HIV Study Group, Yu-Shan Huang, Wang-Da Liu, Guan-Jhou Chen, Wen-Chun Liu, Yi-Ching Su, Pei-Ying Wu, Ling-Ya Chen, Jun-Yu Zhang, Mao-Song Tsai, Chia-Chun Lin, Yu-Lin Lee, Yen-Hsu Chen, Yi-Chia Huang, Wen-Chien Ko, Shu-Hsing Cheng, Sui-Yuan Chang, Ning-Chi Wang, Te-Yu Lin, Yi-Chieh Lee, Shih-Ping Lin, Chia-Yin Hsieh, Hsiu-Wen Wang, Mao-Wang Ho, Tung-Che Hung, Hung-Chin Tsai, Hsi-Hsun Lin, Chen-Hsiang Lee

**Affiliations:** 1 Department of Internal Medicine, National Taiwan University Hospital and National Taiwan University College of Medicine, Taipei, Taiwan; 2 Center of Infection Control, National Taiwan University Hospital, Taipei, Taiwan; 3 Department of Internal Medicine, Far Eastern Memorial Hospital, New Taipei City, Taiwan; 4 School of Medicine, National Yang Ming Chiao Tung University, Taipei, Taiwan; 5 School of Medicine, Chung Shan Medical University, Taichung, Taiwan; 6 Division of Infectious Diseases, Department of Internal Medicine, Chung Shan Medical University Hospital, Taichung, Taiwan; 7 Department of Internal Medicine, Hsinchu MacKay Memorial Hospital, Hsinchu, Taiwan; 8 Department of Internal Medicine, Changhua Christian Hospital, Changhua, Taiwan; 9 Department of Internal Medicine, Kaohsiung Medical University Hospital, 100 Tzyou 1st Road, Kaohsiung, 807 Taiwan; 10 Department of Internal Medicine, Kaohsiung Municipal Ta-Tung Hospital, Kaohsiung, Taiwan; 11 Department of Internal Medicine, National Taiwan University Hospital Hsin-Chu Branch, Hsinchu, Taiwan; 12 Department of Tropical Medicine and Parasitology, National Taiwan University College of Medicine, Taipei, Taiwan; 13 Department of Medicine, College of Medicine, National Cheng Kung University Hospital, National Cheng Kung University, Tainan, Taiwan; 14 Department of Internal Medicine, National Cheng Kung University Hospital, Dou-Liou Branch, College of Medicine, National Cheng Kung University, Yunlin, Taiwan; 15 Department of Internal Medicine, National Taiwan University Hospital Yunlin Branch, Yunlin, Taiwan; 16 Department of Infectious Diseases, Taoyuan General Hospital, Ministry of Health and Welfare, Taoyuan, Taiwan; 17 School of Public Health, National Yang Ming Chiao Tung University, Taipei, Taiwan; 18 College of Medicine, Kaohsiung Medical University, Kaohsiung, Taiwan; 19 Center for Liquid Biopsy and Cohort Research, Kaohsiung Medical University, Kaohsiung, Taiwan; 20 Department of Laboratory Medicine, National Taiwan University Hospital, Taipei, Taiwan; 21 Department of Internal Medicine, Tri-Service General Hospital and National Defense Medical Center, Taipei, Taiwan; 22 Department of Internal Medicine, Lotung Poh-Ai Hospital, Medical Lo-Hsu Foundation, I-Lan, Taiwan; 23 Department of Internal Medicine, Taichung Veterans General Hospital, Taichung, Taiwan; 24 Department of Internal Medicine, China Medical University Hospital, Taichung, Taiwan; 25 Department of Internal Medicine, Ditmanson Medical Foundation Chia-Yi Christian Hospital, Chiayi, Taiwan; 26 Department of Internal Medicine, Kaohsiung Veterans General Hospital, Kaohsiung, Taiwan; 27 Department of Internal Medicine, Kaohsiung Chang Gung Memorial Hospital, Kaohsiung, Taiwan

**Keywords:** Infectious diseases, HIV infections, Tuberculosis

## Abstract

Testing and treatment of tuberculosis infection (TBI) are recommended for people living with HIV (PLWH). We aimed to evaluate the care cascade of TBI treatment among PLWH in the era of antiretroviral therapy (ART) scale-up. This retrospective study included adult PLWH undergoing interferon-gamma release assay (IGRA)-based TBI screening during 2019–2021. PLWH testing IGRA-positive were advised to receive directly-observed therapy for TBI after active TB disease was excluded. The care cascade was evaluated to identify barriers to TBI management. Among 7951 PLWH with a median age of 38 years and CD4 count of 616 cells/mm^3^, 420 (5.3%) tested positive and 38 (0.5%) indeterminate for IGRA. The TBI treatment initiation rate was 73.6% (309/420) and the completion rate was 91.9% (284/309). More than 80% of PLWH concurrently received short-course rifapentine-based regimens and integrase strand transfer inhibitor (InSTI)-containing ART. The main barrier to treatment initiation was physicians’ concerns and patients’ refusal (85.6%). The factors associated with treatment non-completion were older age, female, anti-HCV positivity, and higher plasma HIV RNA. Our observation of a high TBI completion rate among PLWH is mainly related to the introduction of short-course rifapentine-based regimens in the InSTI era, which can be the strategy to improve TBI treatment uptake.

## Introduction

Tuberculosis (TB) is one of the most frequent infectious causes of mortality of people living with HIV (PLWH). Globally, there were an estimated 1.5 million TB deaths in 2020; of those, 14% were attributable to HIV/TB co-infection^1^. HIV infection increases both the risk of progression from primary infection with *Mycobacterium tuberculosis* to active TB disease and reactivation of latent TB^2^. The WHO guidelines therefore strongly recommend systematic testing and treatment of TB infection (TBI) for PLWH^3,4^. Although global progress in TB preventive treatment has been achieved among PLWH, the implementation of targeted testing and treatment of TBI remains suboptimal in Asia^3^. The barriers to optimizing management of TBI include unavailability of tests with better performance, limited access to TBI treatment, and low completion rates with 6 to 9 months of isoniazid regimens^5^.

Compared with 6- and 9-month daily isoniazid monotherapy (6H and 9H, respectively), short-course, rifamycin-based regimens have been shown to have a similar preventive effect with a higher completion rate and lower risk of adverse events^6^. Based on the growing evidence of benefits, the updated guidelines include 3 months of weekly rifapentine plus isoniazid (3HP) as preferred TBI treatment^7^. A clinical trial among PLWH further demonstrated that 1 month of daily rifapentine plus isoniazid (1HP) was also noninferior to the standard 9-month isoniazid for TBI treatment among PLWH who were receiving antiretroviral therapy (ART) consisting predominantly of non-nucleoside reverse-transcriptase inhibitor (NNRTI)-based regimens, but with a higher completion rate^8^.

Rifamycins are potent inducers of hepatic drug-metabolizing enzymes, leading to reduced plasma concentrations of co-administered antiretrovirals^9^. In pharmacokinetic studies, efavirenz or raltegravir can be used with weekly rifapentine without dose adjustment^10,11^; however, clearance of bictegravir (BIC), dolutegravir (DTG), elvitegravir (EVG) and cobicistat (COBI) will be significantly increased when co-administered with rifapentine^12^. Although clinical studies demonstrated that short-course rifapentine-based TBI treatment may not jeopardize the HIV control among PLWH taking BIC- or DTG-containing regimens^13,14^, clinicians may still be reluctant to prescribe rifapentine-based TBI treatment due to the concern of drug interactions^5^.

A previous systematic review of TBI care cascade among PLWH showed that the cumulative proportions of PLWH completing TBI testing and treatment were 83.6 and 41.9%, respectively^15^. Of the included 94,011 PWLH, only 43.6% received ART and 0.3% were treated with short-course rifamycin-based regimens. While ART scale-up has been implemented with the widespread use of integrase strand transfer inhibitor (InSTI) in recent years^12,16^, studies remain limited in PLWH who concurrently receive short-course rifamycin-based regimens and InSTI-containing ART. Taiwan has achieved the Joint United Nations Programme on HIV/AIDS (UNAIDS) 90-90-90 targets, with 90% of PLWH being aware of their HIV status, 94% of those diagnosed being on treatment, and 95% of those on treatment being virally suppressed in 2021. In this study, we aimed to evaluate the cascade of care and identify barriers to the treatment of TBI among PLWH in the era of InSTI-containing ART scale-up.

## Methods

### Study setting and population

Taiwan is a country of intermediate TB burden, with an incidence of 33.2 cases per 100,000 people in 2020^17^. To achieve the WHO End TB Strategy targets, most of the populations at risk for TB identified according to the WHO TBI guidelines have been included for TBI testing and treatment in Taiwan. The national program for TBI has been expanded to include PLWH since 2019 by providing free-of-charge interferon-gamma release assay (IGRA) for PLWH seeking HIV care at the designated hospitals^5,17^. The participants testing positive for IGRA were advised to receive free-of-charge, directly-observed therapy (DOT) for TBI after active TB disease was excluded by review of clinical symptomatology and chest radiography^7^. The available TBI regimens included 1HP, 3HP, 3HR (three months of daily rifampicin plus isoniazid), 4R (four months of daily rifampicin), or 9H^7^. At the end of 2020, the program had been implemented in 33% (26/79) of designated hospitals for HIV care and enrolled 31% (10,563/33,699) of PLWH around Taiwan^18^.

This retrospective cohort study was conducted at 13 major designated hospitals for HIV care in Taiwan. During July 2019 to December 2021, PLWH aged 20 years or more who underwent IGRA-based TBI screening were included. PLWH were excluded from the study if they had had a previous history of TB or TBI treatment. For PLWH with IGRA positivity, eligibility for TBI treatment was evaluated by treating physicians. For those eligible for TBI treatment, TBI treatment regimens were chosen at the discretion of treating physicians. The main treatment considerations included treatment duration, adverse events, and drug interactions. Throughout the treatment course, in-person or smart-phone video-based DOT conducted by HIV case managers was used to monitor treatment adherence and report adverse events^19^. Clinical assessment and laboratory investigations (e.g. complete blood counts, aminotransferases, and bilirubin levels) were followed every 2–4 weeks in the first month and thereafter every 4 weeks until the end of treatment.

HIV medical care, including ART and monitoring of plasma HIV RNA load (PVL) and CD4 count, were provided to all PLWH according to the national HIV treatment guidelines^20^. Though both InSTI- and NNRTI-based regimens are the preferred first-line regimens in Taiwan, the proportion of PLWH initiating InSTI-based regimens increased sharply after the introduction of coformulated single-tablet InSTI-based regimens^16^.

TBI testing and treatment is a national public health policy and all PLWH gave written informed consent to participate in IGRA for TBI. This retrospective study was approved by the Research Ethics Committee or Institutional Review Boards of the participating hospitals (National Taiwan University Hospital [201003112R]; Far Eastern Memorial Hospital [105040-F]; Taoyuan General Hospital [TYGH103011]; Hsinchu Mackay Memorial Hospital [18MMHIS008e]; National Taiwan University Hospital Hsin-Chu Branch [105-017-F]; Chung Shan Medical University Hospital [CS14034]; Changhua Christian Hospital [160408]; National Cheng Kung University Hospital [B-BR-105-038]; Kaohsiung Medical University Hospital [KMUH-IRB20110040]) and informed consent to collection of clinical data for subsequent anonymized analysis was waived. The research was performed in accordance with relevant guidelines and the Declaration of Helsinki^21^.

### TBI care cascade and barriers to TBI treatment

The TBI care cascade evaluated in this study included the following steps: (1) “Medical evaluation completed” as IGRA-positive (defined as interferon-gamma response to TB antigens being significantly above that to negative control) PLWH evaluated by review of clinical symptomatology and chest radiography; (2) “TBI diagnosed” defined as IGRA-positive PLWH without symptoms or image findings suggestive of active TB disease; (3) “Treatment initiated” as IGRA-positive PLWH having been prescribed any TBI treatment regimens; and (4) “Treatment completed” as IGRA-positive PLWH receiving at least 80% of doses within 120% of planned time; and reasons for treatment discontinuation or modification were recorded. The cumulative proportion retained in the care cascade was calculated by multiplying the proportion completing each step by the proportion completing the preceding step. The factors associated with treatment non-initiation and non-completion were analyzed.

### Laboratory investigations

The QuantiFERON-TB Gold In-Tube (QFT-GIT) and QuantiFERON-TB Gold Plus (GFT-Plus) assays (Qiagen, Germantown, MD, USA) were performed by collecting whole blood into separate tubes, which included a Nil tube (negative control), a Mitogen tube (positive control), and one to two TB Antigen tubes^22,23^. IGRA was performed at each participating hospital using the same kit by following the instructions of manufacturer. While the test result was interpreted as positive when the TB Antigen minus Nil value was ≥ 0.35 IU/ml and ≥ 25% of the Nil value, the result was considered indeterminate if the Nil value was > 8.0 IU/mL (high Nil response) or the Mitogen minus Nil value was < 0.5 IU/mL (low Mitogen response). The sensitivity and specificity of QFT assay were 89.0 and 99.1%, respectively.

### Statistical analyses

All statistical analyses were performed with the use of STATA software version 12.0 (Stata Corporation, College Station, TX). Categorical variables were analyzed using the chi-squared test or Fisher’s exact test and continuous variables were compared using the Wilcoxon-Mann–Whitney test. Logistic regression was used to clarify the factors associated with IGRA positivity. Variables with a *P* < 0.05 in univariate analyses were included in the maximum model for multivariate analyses, and a backward selection was used to determine the final model. All statistical tests were two-sided, and variables with *P* < 0.05 were considered significant.

## Results

### Characteristics of PLWH undergoing IGRA testing

**Figure 1:**
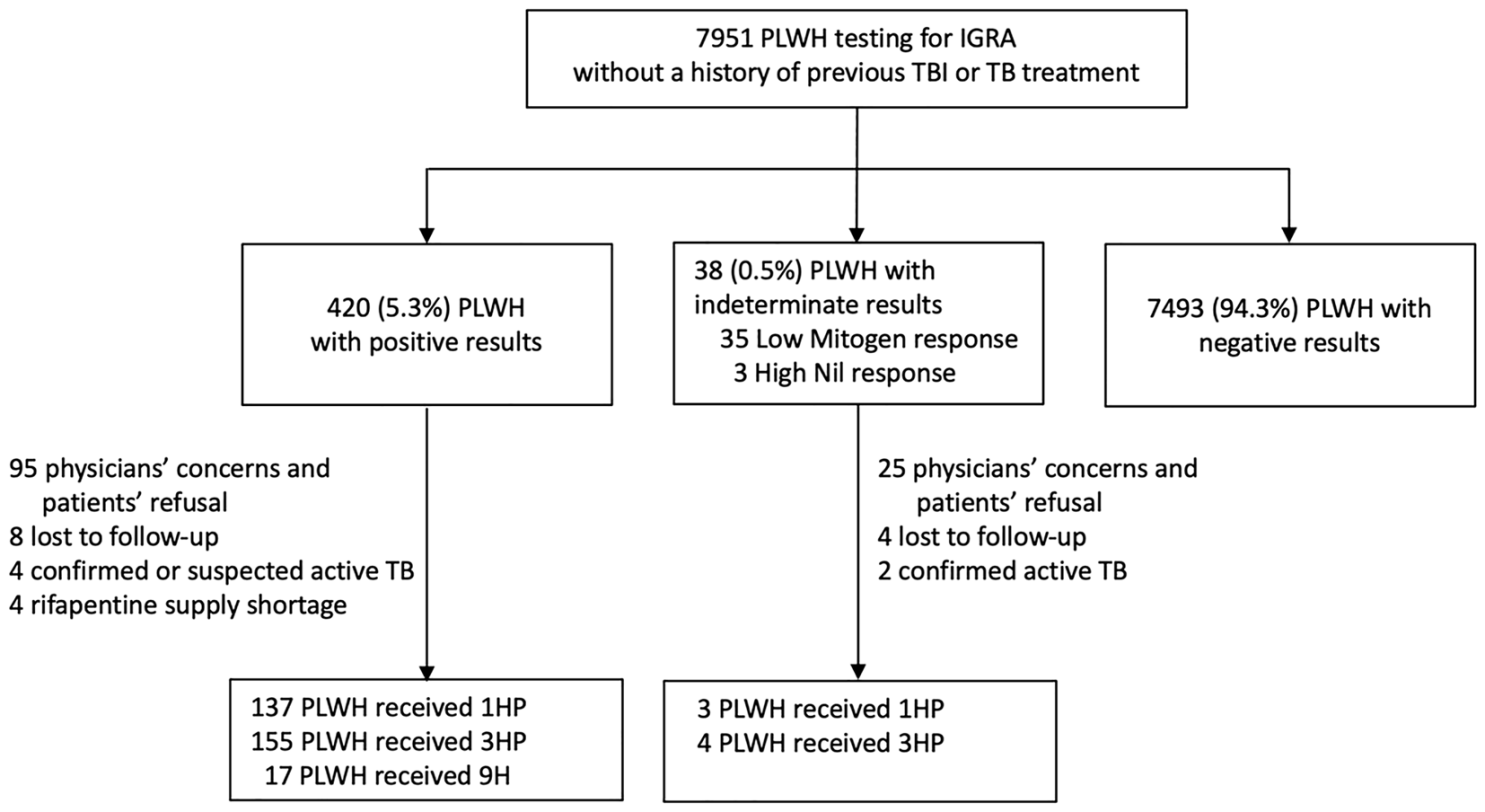
Study flow. 1HP, one month of daily rifapentine plus isoniazid; 3HP, three months of weekly rifapentine plus isoniazid; 9H, nine months of daily isoniazid; TBI, TB infection; IGRA, interferon-gamma release assay; PLWH, people living with HIV; TB, tuberculosis.

From July 2019 to December 2021, a total of 7951 adult PLWH without a previous history of TB or TBI treatment underwent IGRA testing (Fig. 1); the majority of them were male (96.0%) with a median age of 38 years (interquartile range [IQR], 31–45). The main route of HIV transmission was male-to-male sexual contact (79.5%), followed by illicit drug use (11.6%) and heterosexual sexual contact (6.9%). A high proportion (96.4%) of enrolled PLWH had achieved PVL < 200 copies/mm^3^ and the median CD4 count was 616 cells/mm^3^.

Overall, 420 (5.3%) tested positive for IGRA and 38 (0.5%) had indeterminate results. Compared with PLWH with IGRA negativity, those with IGRA positivity were older (median age, 43 vs. 38 years) and less likely to be male (92.4 vs. 96.2%). IGRA-positive PLWH were more likely to have acquired HIV through heterosexual contact (11.7 vs. 6.6%) and illicit drug use (25.0 vs. 10.8%), and to have had a history of incarceration (9.1 vs. 5.8%), anti-HCV positivity (28.3 vs. 16.2%), and higher baseline CD4 counts (median, 639 vs. 615 cells/mm^3^) (all *P* values < 0.05) (Table 1). In multivariable analysis, the independent factors associated with IGRA positivity were older age (per 1-year increase, adjusted odds ratio [AOR], 1.04; 95% confidence interval [CI], 1.02–1.05), illicit drug use (AOR, 2.11; 95% CI, 1.49–3.00), and higher baseline CD4 counts (per 10-cell/mm^3^ increase, AOR, 1.01; 95% CI, 1.00–1.01) (Table 2).

**Table 1:** Clinical characteristics of PLWH with positive and negative IGRA results.

	Positive IGRA results (n = 420)	Negative IGRA results (n = 7493)	*P* value
Age, median (IQR), years	43 (35–51)	38 (31–45)	< 0.001
Male sex, n (%)	388 (92.4)	7209 (96.2)	< 0.001
**Risk group for HIV transmission, n (%)**			
Men who have sex with men	256 (61.0)	6036 (80.6)	< 0.001
Heterosexuals	49 (11.7)	493 (6.6)	< 0.001
Illicit drug users	105 (25.0)	810 (10.8)	< 0.001
Others or unknown	10 (2.4)	154 (2.1)	0.648
History of incarceration, n (%)	38 (9.1)	432 (5.8)	0.006
**Comorbidity, n (%)**			
HBsAg positivity	47 (11.2)	730 (9.7)	0.332
Anti-HCV positivity	119 (28.3)	1210 (16.2)	< 0.001
Cardiovascular disease	26 (6.2)	462 (6.2)	0.984
Cerebrovascular disease	1 (0.2)	8 (0.1)	0.437
Diabetes mellitus	16 (3.8)	228 (3.0)	0.376
Chronic kidney disease^a^	2 (0.5)	27 (0.4)	0.702
Undergoing dialysis	0 (0)	5 (0.1)	0.596
Chronic obstructive pulmonary disease or asthma	1 (0.2)	16 (0.2)	0.916
Malignancy	2 (0.5)	13 (0.2)	0.165
Autoimmune disease	0 (0)	9 (0.1)	0.477
Receiving immunosuppressive therapy^b^	2 (0.5)	9 (0.1)	0.057
CD4 count at screening, median (IQR), cells/mm^3^	639 (482–831)	615 (453–802)	0.022
PVL at screening, median (range), log_10_ copies/mL	UD (UD-6.06)^c^	UD (UD-6.88)	0.161

*HBsAg* Hepatitis B surface antigen, *HCV* Hepatitis C virus, *IGRA* Interferon-gamma release assay, *IQR* Interquartile range, *TBI* Tuberculosis infection, *PLWH* People living with HIV, *PVL* Plasma HIV RNA load, *UD* Undetectable.

^a^Chronic kidney disease was defined as reduced glomerular filtration rate or kidney damage (< 60 ml/min/1.73 m^2^ of body-surface area) for more than 3 months.

^b^Immunosuppresive therapy included chemotherapy, corticosteroids, and biologic agents.

^c^UD, < 50 copies/mL.

**Table 2:** Factors associated with positive IGRA results.

	Univariable analysis		Multivariable analysis	
	OR (95% CI)	*P* value	OR^a^ (95% CI)	*P* value
Age, per 1-year increase	1.04 (1.03–1.05)	< 0.001	1.04 (1.02–1.05)	< 0.001
Male sex	0.48 (0.33–0.70)	< 0.001	0.94 (0.61–1.45)	0.780
**Risk group for HIV transmission**				
Men who have sex with men	0.38 (0.31–0.46)	< 0.001	Reference	
Heterosexuals	1.88 (1.37–2.56)	< 0.001	1.45 (0.98–2.13)	0.063
Illicit drug users	2.75 (2.18–3.47)	< 0.001	2.11 (1.49–3.00)	< 0.001
Others or unknown	1.16 (0.61–2.22)	0.649		
History of incarceration	1.63 (1.15–2.30)	0.006	0.96 (0.63–1.45)	0.842
HBsAg positivity	1.17 (0.85–1.60)	0.332		
Anti-HCV positivity	2.05 (1.65–2.56)	< 0.001	1.25 (0.93–1.68)	0.139
Cardiovascular disease	1.00 (0.67–1.51)	0.984		
Cerebrovascular disease	2.23 (0.28–17.90)	0.449		
Diabetes mellitus	1.26 (0.75–2.12)	0.378		
Chronic kidney disease^b^	1.32 (0.31–5.58)	0.703		
Receiving dialysis	3.57 (0.42–30.66)	0.245		
Chronic obstructive pulmonary disease or asthma	1.12 (0.15–8.43)	0.916		
Malignancy	2.75 (0.62–12.24)	0.183		
Autoimmune disease	1.98 (0.25–15.70)	0.516		
Receiving immunosuppressive therapy^c^	3.98 (0.86–18.47)	0.078		
CD4 count at screening, per 10-cell/mm^3^ increase	1.00 (1.00–1.01)	0.006	1.01 (1.00–1.01)	< 0.001
PVL at screening, per 1-log_10_ copies/mL increase	0.89 (0.78–1.02)	0.098		

*CI* Confidence interval, *HBsAg* Hepatitis B surface antigen, *HCV* Hepatitis C virus, *IGRA* Interferon-gamma release assay, *OR* Odds ratio, *PLWH* People living with HIV, *PVL* Plasma HIV RNA load.

^a^The ORs are the estimates of the effect of covariates on IGRA positivity, adjusted for age, sex, transmission routes, history of incarceration, anti-HCV positivity, and CD4 count at screening using logistic regression analysis.

^b^Chronic kidney disease was defined as reduced glomerular filtration rate or kidney damage (< 60 ml/min/1.73 m^2^ of body-surface area) for more than 3 months.

^c^Immunosuppressive therapy included chemotherapy, corticosteroids, and biologic agents.

Of 38 PLWH with indeterminate IGRA results, 35 PLWH had low mitogen responses and 3 had high nil responses. PLWH with low mitogen responses had significantly lower baseline CD4 counts (median, 414 cells/mm^3^) compared to PLWH with positive and those with negative IGRA results (median, 639 and 615 cells/mm^3^, respectively) (Supplementary Table 1).

### TBI treatment initiation

Overall, 73.6% (309/420) and 18.4% (7/38) of PLWH with positive IGRA and those with indeterminate IGRA results, respectively, initiated TBI treatments (Fig. 1). Of those IGRA-positive not receiving TBI treatment, the common reasons for not initiating TBI treatment were physicians’ concerns about adverse effects and drug-drug interactions and patients’ refusal (95/111, 85.6%) and loss to follow-up (8/111, 7.2%). However, there was no specific demographic variable independently predicting non-initiation (Supplementary Tables 3 and 4). Among 309 IGRA-positive PLWH initiating TBI treatment, the most common TBI regimens were 3HP (155/309, 50.2%), followed by 1HP (137/309, 44.3%) and 9H (17/309, 5.5%). The most frequent ART used was DTG-based regimens (166/309, 53.7%) and coformulated BIC, emtricitabine and tenofovir alafenamide (BIC/FTC/TAF) (105/309, 34.0%) (Supplementary Table 4).

### TBI treatment completion

The overall completion rate was 91.9% (284/309): 93.4% in 137 PLWH receiving 1HP, 89.7% in 155 PLWH receiving 3HP, and 100% in 17 PLWH receiving 9H (Supplementary Table 4). Among 25 PLWH who failed to complete TBI treatment, 23 (92.0%) were due to adverse events and 2 (8.0%) due to drug interactions with methadone. The most common reasons of treatment non-completion included abnormalities in liver function tests (total bilirubin, 0.86–4.09 mg/dL; and alanine aminotransferase, 48–472 U/L) (6/29, 20.7%), urticaria (6/29, 20.7%), and nausea (6/29, 20.7%). The median time to discontinuation was 14 days (95% CI, 12–21 days). Compared with PLWH with treatment completion, PLWH with treatment non-completion were older (median age, 49 vs. 42 years), less likely to be male (76.0% vs. 94.4%), and more likely to have acquired HIV through illicit drug use (36.0 vs. 19.7%), have anti-HCV positivity (40.0 vs. 11.6%), and receive DTG-containing regimens (80.0 vs. 51.4%) (Supplementary Table 2). In multivariable analysis, the predictors of treatment non-completion were older age (per 1-year increase, AOR, 1.07; 95% CI, 1.02–1.11), not male sex (AOR for male, 0.27; 95% CI, 0.09–0.85), anti-HCV positivity (AOR, 4.45; 95% CI, 1.73–11.47), and PVL before TBI treatment (per 1-log_10_ copies/mL increase, AOR, 1.78; 95% CI, 1.07–2.95) (Table 3).

**Table 3:** Factors associated with treatment non-completion.

Variable	Univariable analysis		Multivariable analysis	
	OR (95% CI)	*P* value	OR^a^ (95% CI)	*P* value
**TBI regimen**				
9H	Reference	–		
1HP	1.51 (0.41–5.60)	0.540		
3HP	2.12 (0.84–5.36)	0.114		
Age, per 1-year increase	1.06 (1.02–1.10)	0.001	1.07 (1.02–1.11)	0.002
Male sex	0.19 (0.07–0.54)	0.002	0.27 (0.09–0.85)	0.025
**Risk group for HIV transmission**				
Men who have sex with men	Reference	–		
Heterosexuals	1.51 (0.41–5.60)	0.540		
Illicit drug users	2.12 (0.84–5.36)	0.114		
Body-mass index, per 1-kg/m^2^ increase	1.09 (0.95–1.26)	0.218		
History of incarceration	0.47 (0.06–3.66)	0.473		
HBsAg positivity	1.90 (0.52–6.93)	0.330		
Anti-HCV positivity	5.07 (2.11–12.21)	< 0.001	4.45 (1.73–11.47)	0.002
Cardiovascular disease	0.47 (0.06–3.66)	0.473		
Diabetes mellitus	0.80 (0.10–6.38)	0.836		
**ART during TBI treatment**				
Bictegravir-containing regimen	1.47 (0.16–13.54)	0.736		
Dolutegravir-containing regimen	5.07 (0.66–39.90)	0.119		
Others	Reference	–		
CD4 count before TBI treatment, per 10-cells/mm^3^ increase	0.99 (0.98–1.01)	0.200		
PVL before TBI treatment, per 1-log_10_ copies/mL increase	1.90 (1.19–3.03)	0.007	1.78 (1.07–2.95)	0.026

*1HP* One month of daily rifapentine plus isoniazid, *3HP* Three months of weekly rifapentine plus isoniazid, *ART* Combination antiretroviral therapy, *HBsAg* Hepatitis B surface antigen, *HCV* Hepatitis C virus, *TBI* Tuberculosis infection, *OR* Odds ratio, *PVL* Plasma HIV RNA load.

^a^For the estimates of the effect of covariates on treatment noncompletion, the ORs are adjusted for age, sex, anti-HCV positivity, and PVL before TBI treatment using logistic regression analysis.

### TBI care cascade

**Figure 2:**
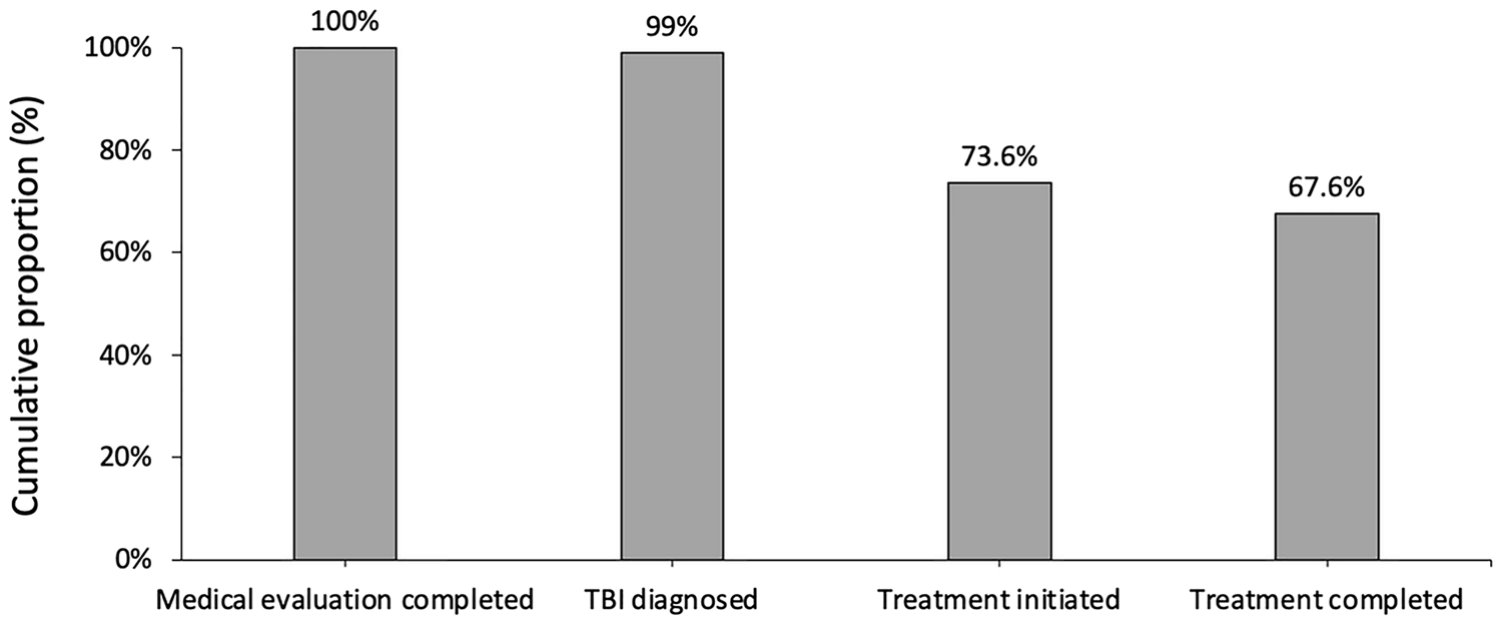
Cascade of care in TBI treatment among IGRA-positive PLWH. TBI, TB infection; PLWH, people living with HIV.

Among IGRA-positive PLWH, the cumulative proportions of medical evaluation completed, TBI diagnosed, treatment initiated, and treatment completed were subsequently 100% (420/420), 99.0% (416/420), 73.6% (99.0% × [309/416]) and 67.6% (73.6% × [284/309]), respectively (Fig. 2). The main gap of care cascade occurred at the step of treatment initiation (25.7%, 107/416).

## Discussion

In this study conducted during the roll-out of TBI screening and treatment in Taiwan, we found the overall prevalence of TBI was 5.3% among PLWH in the modern era of ART and declining TB incidence. The TBI treatment uptake and completion rates in this cohort were 73.6 and 91.9%, respectively, when short-course rifamycin-based regimens available. More than 80% of the included PLWH were concurrently receiving short-course rifapentine-based regimens and InSTI-containing ART and the major gap of engagement in care was related to TBI treatment initiation.

Targeted testing and treatment for TBI have been prioritized for PLWH and showed cost-effectiveness in countries with different TB endemicities^24,25^. The United States reported the lowest TB incidence rate in the world (2.7 per 100,000 population in 2019) and estimated an TBI prevalence rate to be 4.7% among 1510 PLWH^26^. Hong Kong, as a city with intermediate TB burden, had an TBI prevalence of 26.2% among 2412 PLWH^27^. In Taiwan, a previous study including 909 PLWH during 2008–2010 demonstrated that the TBI prevalence was 36.6% in men who have sex with men (MSM), 12.7% in heterosexuals, and 50.7% in injecting drug users^28^. Another study of 608 PLWH who were mainly MSM during 2011–2013 found a lower TBI prevalence (10.5%)^29^. Compared with the PLWH included in the earlier study (median CD4, 441–578 cells/mm^3^; proportion of PVL < 50 copies/mm^3^, 50.0%), our study cohort in the era of ART scale-up had improved immunologic and virologic characteristics (median CD4, 616 cells/mm^3^; proportion of PVL < 50 copies/mm^3^, 92.2%)^29^, which could reduce the probability of both false-negative and indeterminate IGRA results^30^. Therefore, the even lower TBI prevalence shown in our study might reflect the steadily declining TB incidence in Taiwan^17^. The findings of association of older age, illicit drug use, and increased CD4 counts with positive TBI test results in this study are in line with those in other studies^3,29,31^.

In a recent meta-analysis, the estimated rates of TBI treatment initiation and completion among PLWH with positive TBI tests were 86.3 and 69.4%, respectively. The main losses of the care cascade were in the provider recommendation and TBI treatment completion. The reported barriers included adverse events, pill burden, drug interactions, and lack of knowledge among healthcare workers and patients. In the 70 included cohorts, only one primarily used rifamycin-based short-course regimens (3–4 months of rifampicin plus isoniazid)^15^. In general population, short-course preventive therapy regimens facilitated treatment uptake and enhance treatment completion for individuals with TBI^6^. The extension of PREVENT TB trial enrolled 399 PLWH and showed a higher completion rate of 3HP when compared with 9H (89 vs. 64%)^32^. The BRIEF TB trial that enrolled 3000 PLWH mainly receiving NNRTI-based antiretroviral therapy also demonstrated a higher completion rate of 1HP compared with 9H (97 vs. 90%)^8^. While the treatment completion rate in our cohort study was consistent with those observed in the clinical trial settings^8,32^, the findings of factors identified to be associated with treatment non-completion imply the need for carefully monitoring adverse events in older PLWH, and those with viral hepatitis and virological non-suppression. Individuals with older age and viral hepatitis are more likely to develop adverse drug reactions during TBI treatment, particularly hepatotoxicity. Virological non-suppression could be related to poor linkage to care and adherence to medicine, resulting in treatment non-completion. Therefore, treatment completion may be enhanced by counseling, support, and comprehensive follow-up.

Despite the adoption of short-course rifapentine-based regimens during the launch of TBI testing and treatment program, the treatment initiation rate in our study (73.6%) was still lower than that in previous studies^15^. While co-formulated single-tablet InSTI-containing regimens have become the recommended first-line HIV treatment regimens^12^, concerns about adverse effects and drug-drug interactions cause the hesitancy to initiate TBI treatment in this cohort. Indeed, co-administration of rifapentine significantly decreases the plasma concentrations of InSTIs due to rifapentine-mediated CYP3A and UGT1A1 induction^33^. In a phase 1/2 trial conducted among 61 PLWH concurrently receiving DTG-containing regimens and 3HP, a 36% increase in DTG clearance was observed; however, only one had trough concentration below the 90% maximal inhibitory concentration for DTG and all participants were able to maintain HIV viral suppression during 3HP treatment^13^. In another study of 48 PLWH concurrently receiving BIC/FTC/TAF and 1HP, the proportion of BIC trough concentrations above the 95% effective concentration dropped to 37%; however, more than 90% of participants were able to maintain HIV viral suppression during and after 1HP treatment^14^. Although the findings suggest that viral suppression still could be maintained and no cases of virologic failure occurred after TBI treatment among PLWH receiving InSTI-containing regimens, transient HIV viremia was noted during TBI treatment due to a significant decrease in BIC trough concentration. Considering that viral blips are not predictive of virologic failure^12,34^, both BIC- and DTG-containing regimens could be considered to be co-administered with 1HP or 3HP under virologic monitoring, though more clinical and pharmacokinetic studies are warranted to confirm the findings.

Our study has several limitations. First, the study was conducted among 23.6% (7,951/33,699) of PLWH around Taiwan; therefore, the prevalence and associated factors of TBI reported in this study may not be generalized to a larger population. Second, the absence of clinical symptoms or radiographic abnormalities may not exclude incipient and subclinical TB. Furthermore, the indeterminate IGRA results cannot provide useful information about the likelihood of *M. tuberculosis* infection, but only a few PLWH with indeterminate results underwent repeat IGRA tests till the end of the study. Lastly, the detailed reasons for not considering to initiate TBI treatment by both IGRA-positive PLWH or care providers could not be clearly defined in this retrospective cohort study and concerns might differ across physicians. More qualitative studies are needed to better understand these concerns and more investigations on the safety profile and maintenance of viral suppression among PLWH receiving TBI treatment are needed to alleviate the concerns.

## Conclusions

The high completion rate among those mainly receiving short-course rifapentine-based regimens in the InSTI era indicated the current strategy to be effective in improving the TBI treatment for PLWH.

## Supplementary Information

Supplementary Information.

## Data Availability

The datasets generated and/or analyzed during the current study are not publicly available, since the participants did not consent to the sharing of data with third parties, but are available from the corresponding author on reasonable request.
