# Supplementary material for: Care cascade of tuberculosis infection treatment for people living with HIV in the era of antiretroviral therapy scale-up
Source: Sci Rep. 2022 Sep 27;12:16136. doi: 10.1038/s41598-022-20394-2 (PMC9515204; doi:10.1038/s41598-022-20394-2)
Supplement: Supplementary file 1 — Supplementary Information. [file 41598_2022_20394_MOESM1_ESM.docx]

**Supplementary Material**

Supplement to:

**Care cascade of tuberculosis infection treatment for people living with HIV in the era of antiretroviral therapy scale-up**

Kuan-Yin Lin, Chia-Jui Yang, Hsin-Yun Sun, Yuan-Ti Lee, Bo-Huang Liou, Ing-Moi Hii, Tun-Chieh Chen, Sung-Hsi Huang, Chun-Yuan Lee, Chin-Shiang Tsai, Chi-Ying Lin, Chun-Eng Liu, Hsi-Yen Chang, Chien-Yu Cheng, Po-Liang Lu, Chien-Ching Hung, on behalf of the Taiwan HIV Study Group

Content:

Table S1. Clinical characteristics of 38 PLWH with indeterminate IGRA results

Table S2. Clinical characteristics of PLWH initiating and not initiating TBI treatment

Table S3. Factors associated with TBI treatment non-initiation

Table S4. Clinical characteristics of PLWH completing and not completing TBI treatment

**Table S1. Clinical characteristics of 38 PLWH with indeterminate IGRA results**

|  | **Low Mitogen response**  **(n=35)** | **High Nil response (n=3)** | ***P* value** |
| --- | --- | --- | --- |
| Age, median (IQR), years | 39 (31-45) | 45 (29-53) | 0.533 |
| Male sex, n (%) | 31 (88.6) | 3 (100.0) | 0.536 |
| Transmission route, n (%) |  |  |  |
| Men who have sex with men | 24 (68.6) | 3 (100.0) | 0.249 |
| Heterosexuals | 5 (14.3) | 0 (0) | 0.482 |
| Illicit drug use | 6 (17.1) | 0 (0) | 0.435 |
| History of incarceration, n (%) | 2 (5.7) | 0 (0) | 0.671 |
| Comorbidity, n (%) |  |  |  |
| HBsAg positivity | 4 (11.4) | 0 (0) | 0.536 |
| Anti-HCV positivity | 5 (14.3) | 0 (0) | 0.482 |
| Cardiovascular disease | 2 (5.7) | 0 (0) | 0.671 |
| Diabetes mellitus | 2 (5.7) | 0 (0) | 0.671 |
| CD4 count at screening, median (IQR), cells/mm^3^ | 414 (230-723) | 681 (521-1030) | 0.088 |
| PVL at screening, median (range), log_10_ copies/mL | UD (UD-5.30)^a^ | UD (UD-UD) | 0.189 |

Abbreviations: HBsAg, hepatitis B surface antigen; HCV, hepatitis C virus; IGRA, interferon-gamma release assay; IQR, interquartile range; PLWH, people living with HIV; PVL, plasma HIV RNA load; UD, undetectable.

^a^UD, <20 copies/mL.

**Table S2. Clinical characteristics of PLWH initiating and not initiating TBI treatment**

|  | **Treatment initiation (n=309)** | **Treatment non-initiation (n=111)** | ***P* value** |
| --- | --- | --- | --- |
| Age, median (IQR), years | 42 (35-50) | 47 (36-54) | 0.006 |
| Male sex, n (%) | 286 (92.6) | 102 (91.9) | 0.821 |
| Risk group for HIV transmission, n (%) |  |  |  |
| Men who have sex with men | 207 (67.0) | 49 (44.1) | <0.001 |
| Heterosexuals | 34 (11.0) | 15 (13.5) | 0.480 |
| Illicit drug users | 65 (21.0) | 40 (36.0) | 0.002 |
| Others or unknown | 3 (1.0) | 7 (6.3) | 0.002 |
| History of incarceration, n (%) | 23 (7.4) | 15 (13.5) | 0.056 |
| Comorbidity, n (%) |  |  |  |
| HBsAg positivity | 30 (9.7) | 17 (15.3) | 0.108 |
| Anti-HCV positivity | 75 (24.3) | 44 (39.6) | 0.002 |
| Cardiovascular disease | 19 (6.2) | 7 (6.3) | 0.953 |
| Cerebrovascular disease | 1 (0.3) | 0 (0) | 0.548 |
| Diabetes mellitus | 13 (4.2) | 3 (2.7) | 0.478 |
| Chronic kidney disease^a^ | 2 (0.7) | 0 (0) | 0.396 |
| Chronic obstructive pulmonary disease or  asthma | 1 (0.3) | 0 (0) | 0.548 |
| Malignancy | 2 (0.7) | 0 (0) | 0.396 |
| Receiving immunosuppressive therapy^b^ | 2 (0.7) | 0 (0) | 0.396 |
| CD4 count at screening, median (IQR), cells/mm^3^ | 645 (503-832) | 611 (441-812) | 0.298 |
| PVL at screening, median (range), log_10_ copies/mL | UD (UD-2.98)^c^ | UD (UD-6.06) | 0.118 |

Abbreviations: HBsAg, hepatitis B surface antigen; HCV, hepatitis C virus; IGRA, interferon-gamma release assay; IQR, interquartile range; TBI, tuberculosis infection; PLWH, people living with HIV; PVL, plasma HIV RNA load; UD, undetectable.

^a^Chronic kidney disease was defined as reduced glomerular filtration rate or kidney damage (<60 ml/min/1.73 m^2^ of body-surface area) for more than 3 months.

^b^Immunosuppresive therapy included chemotherapy, corticosteroids, and biologic agents.

^c^UD, <50 copies/mL.

**Table S3. Factors associated with TBI treatment non-initiation**

|  | **Univariable analysis** | | **Multivariable analysis** | |
| --- | --- | --- | --- | --- |
|  | **OR (95% CI)** | ***P* value** | **OR^a^ (95% CI)** | ***P* value** |
| Age, per 1-year increase | 1.03 (1.01-1.05) | 0.011 | 1.01 (0.99-1.03) | 0.410 |
| Male sex | 0.91 (0.41-2.03) | 0.821 |  |  |
| Risk group for HIV transmission |  |  |  |  |
| Men who have sex with men | 0.39 (0.25-0.61) | <0.001 | Reference |  |
| Heterosexuals | 1.26 (0.66-2.42) | 0.481 | 1.71 (0.82-3.58) | 0.154 |
| Illicit drug users | 2.11 (1.32-3.40) | 0.002 | 2.09 (0.99-4.41) | 0.054 |
| Others or unknown | 6.87 (1.74-27.04) | 0.006 | 8.22 (1.99-33.90) | 0.004 |
| History of incarceration | 1.94 (0.97-3.87) | 0.059 |  |  |
| HBsAg positivity | 1.68 (0.89-3.19) | 0.111 |  |  |
| Anti-HCV positivity | 2.05 (1.29-3.25) | 0.002 | 1.22 (0.61-2.42) | 0.573 |
| Cardiovascular disease | 1.03 (0.42-2.51) | 0.953 |  |  |
| Diabetes mellitus | 0.63 (0.18-2.26) | 0.481 |  |  |
| CD4 count at screening, per 10-cell/mm^3^ increase | 1.00 (0.99-1.00) | 0.549 |  |  |
| PVL at screening, per 1-log_10_ copies/mL increase | 1.38 (1.04-1.82) | 0.025 | 1.29 (0.96-1.73) | 0.087 |

Abbreviations: CI, confidence interval; HBsAg, hepatitis B surface antigen; HCV, hepatitis C virus; IGRA, interferon-gamma release assay; OR, odds ratio; PLWH, people living with HIV; PVL, plasma HIV RNA load.

^a^The ORs are the estimates of the effect of covariates on treatment non-initiation, adjusted for transmission routes, anti-HCV positivity, and PVL at screening using logistic regression analysis.

^b^Chronic kidney disease was defined as reduced glomerular filtration rate or kidney damage (<60 ml/min/1.73 m^2^ of body-surface area) for more than 3 months.

^c^Immunosuppressive therapy included chemotherapy, corticosteroids, and biologic agents.

**Table S4. Clinical characteristics of PLWH completing and not completing TBI treatment**

|  | **Treatment completion**  **(n=280)** | **Treatment** **non-completion**  **(n=29)** | ***P* value** |
| --- | --- | --- | --- |
| TBI regimen, n (%) |  |  |  |
| 1HP | 127 (45.4) | 10**^a^** (34.5) | 0.262 |
| 3HP | 136 (48.6) | 19^b^ (65.5) | 0.082 |
| 9H | 17 (6.1) | 0 (0) | 0.172 |
| Age, median (IQR), years | 42 (35-50) | 49 (42-55) | <0.001 |
| Male sex, n (%) | 264 (94.3) | 23 (79.3) | 0.003 |
| Transmission route, n (%) |  |  |  |
| Men who have sex with men | 197 (70.4) | 12 (41.4) | 0.001 |
| Heterosexuals | 29 (10.4) | 4 (13.8) | 0.568 |
| Illicit drug use | 53 (18.9) | 12 (41.4) | 0.005 |
| Body-mass index, median (IQR), kg/m^2^ | 23.8 (21.6-26.2) | 24.9 (22.2-26.4) | 0.291 |
| History of incarceration, n (%) | 22 (7.9) | 2 (6.9) | 0.854 |
| Comorbidity, n (%) |  |  |  |
| HBsAg positivity | 19 (6.8) | 3 (10.3) | 0.478 |
| Anti-HCV positivity | 31 (11.1) | 12 (41.4) | <0.001 |
| Cardiovascular disease | 22 (7.9) | 2 (6.9) | 0.854 |
| Cerebrovascular disease | 1 (0.4) | 0 (0) | 0.747 |
| Diabetes mellitus | 14 (5.0) | 1 (3.5) | 0.711 |
| Chronic kidney disease^c^ | 1 (0.4) | 0 (0) | 0.747 |
| Chronic obstructive pulmonary disease or asthma | 2 (0.7) | 0 (0) | 0.648 |
| Malignancy | 2 (0.7) | 0 (0) | 0.648 |
| ART during TBI treatment |  |  |  |
| Bictegravir-containing regimen | 101 (36.1) | 4 (13.8) | 0.016 |
| Dolutegravir-containing regimen | 143 (51.1) | 23 (79.3) | 0.004 |
| Others | 36 (12.9) | 2 (6.9) | 0.352 |
| CD4 count before TBI treatment, median (IQR), cells/mm^3^ | 645 (504-831) | 550 (471-741) | 0.096 |
| PVL before TBI treatment, median (range), log_10_ copies/mL | UD (UD-2.98)^d^ | UD (UD-4.74) | 0.051 |

Abbreviations: 1HP, one month of daily rifapentine plus isoniazid; 3HP, three months of weekly rifapentine plus isoniazid; 9H, nine months of daily isoniazid; ART, antiretroviral therapy; HBsAg, hepatitis B surface antigen; HCV, hepatitis C virus; TBI, tuberculosis infection; IQR, interquartile range; PLWH, people living with HIV; PVL, plasma HIV RNA load; UD, undetectable.

^a^In 1HP group, 9 PLWH discontinued due to abnormalities in liver function tests (n=5), urticaria (2), fever (1), myalgia (1), headache (1), diarrhea (1), mood alteration (1), and drug interaction with methadone (1). One PLWH was switched to 3HP due to fatigue and dizziness.

^b^In 3HP group, 16 PLWH discontinued 3HP due to nausea (5), anorexia (3), dizziness (3), urticaria (3), fever (1), headache (1), fatigue (1), abdominal discomfort (1), diarrhea (1), abnormalities in liver function tests (1), and stroke (1). Two PLWH were switched to 9H due to nausea, myalgia, and fatigue (n=1) as well as drug interactions with methadone (1). One PLWH was switched to 3HR due to insomnia and pruritus.

^c^Chronic kidney disease was defined as reduced glomerular filtration rate or kidney damage (<60 ml/min/1.73 m^2^ of body-surface area) for more than 3 months.

^d^UD, <50 copies/mL.
